# SARS-CoV-2 Omicron subvariant BA.2.86: limited potential for global spread

**DOI:** 10.1038/s41392-023-01712-0

**Published:** 2023-11-30

**Authors:** Xinling Wang, Lu Lu, Shibo Jiang

**Affiliations:** Shanghai Institute of Infectious Disease and Biosecurity, MOE/NHC/CAMS Key Laboratory of Medical Molecular Virology, Shanghai Frontiers Science Center of Pathogenic Microorganisms and Infection, School of Basic Medical Sciences, Shanghai Medical College, Fudan University, Shanghai, China

**Keywords:** Infectious diseases, Infectious diseases

In recent studies published in *Nature* and *Lancet Infectious Diseases*,^1,2^ SARS-CoV-2 Omicron subvariant BA.2.86 exhibited a substantial antigenic drift, enhanced receptor affinity, and less immune evasion to sera of individuals upon XBB breakthrough infection or reinfection. These findings suggest that the potential for BA.2.86 to spread globally is limited, albeit the mutated BA.2.86 sequences were detected in wastewater in various public and commercial venues before the confirmed case of BA.2.86 infection in Thailand, which raised concerns about the spread of BA.2.86 through international travel.^3^

**Fig. 1:**
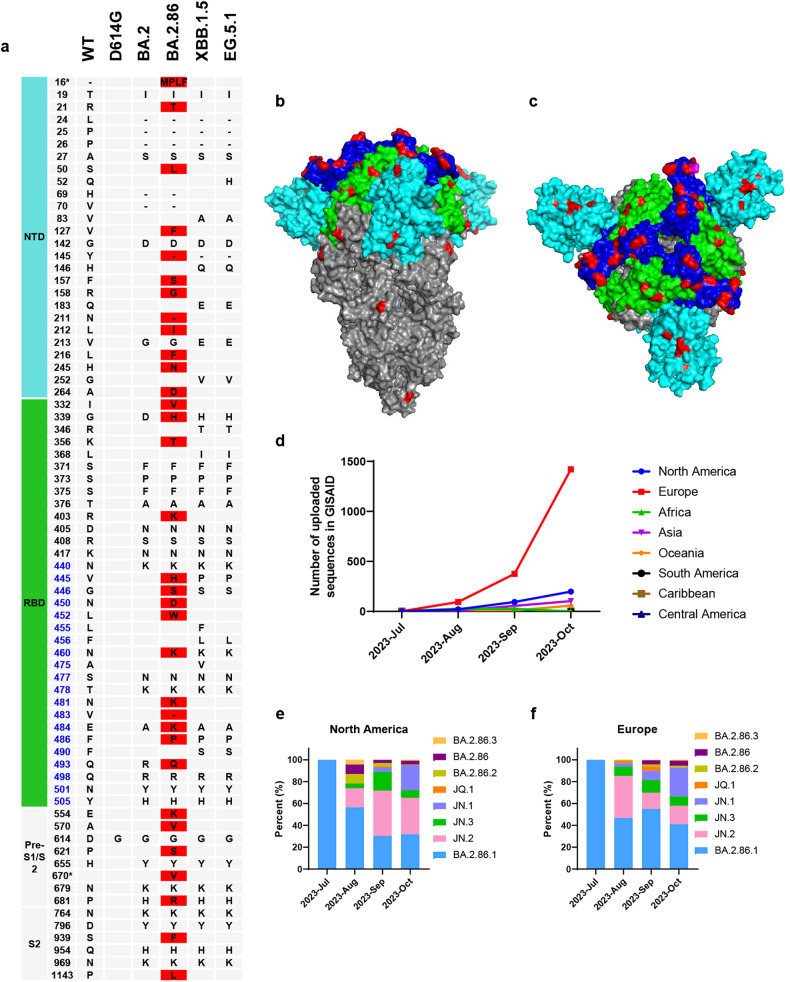
The mutated amino acids in S protein of BA.2.86 and its global prevalence. **a** The mutated amino acids in S proteins of BA.2.86 different from those of SARS-CoV-2 wildtype (WT), D614G, BA.2, XBB.1.5 and EG.5.1. Different amino acids in BA.2.86 compared with BA.2 were colored in red. *I670V mutation was found in the sequence uploaded by Levytskyi and Katya in Israel (accession number: EPI_ISL_18096761). **b**, **c** The crystal structure of BA.2 S protein on closed conformation. The NTD, RBD and RBM areas were colored cyan, green and blue, respectively. The mutated amino acids in S protein of BA.2.86 different from those in BA.2 were colored red. Protein Data Bank accession code: 7XIX. **d** Number of uploaded BA.2.86 sequences in GISAID that collected in different regions of the world from July 2023 to October 2023. **e**, **f** Percentage of different sublineages of BA.2.86 in North America (**e**) and Europe (**f**) from July to October 2023

BA.2.86, a novel lineage of the Omicron variant known as the “second generation of BA.2”, was first detected in Denmark and Israel in late July 2023. It was subsequently seen in several regions worldwide, including Australia, the United Kingdom, France, Canada, and the United States. On August 17, 2022, WHO classified BA.2.86 as a “variant under monitoring”. It bears 33 and over 35 spike mutations that differ from those in BA.2 and XBB.1.5, respectively (Fig. 1a). Using the crystal structure of BA.2 as a reference, these novel mutation sites in BA.2.86 are mainly located in the N-terminal domain (NTD) and the receptor-binding domain (RBD) regions in S1 subunit of S protein (Fig. 1b, c), leading to the alteration of receptor-binding and immune-evasion capability. Among these mutations, P681R seems to boost S protein functions. For example, spike-mediated virus-cell membrane fusion might be improved, while K356T, N450D, L452W, A445H, A484K, and V483del are related to enhanced antibody resistance compared with XBB.1.5.^1^

Researchers evaluated the cellular infectivity of BA.2.86 by testing the pseudovirus entry into target cells, including Vero cells, and human ACE2-HEK293T cells.^1^ The results showed that BA.2.86 exhibited lower infectivity than that of XBB.1.5, EG.5, and HK.3, but showed an ACE2-biding affinity (K_D_ value) about 3- and 2.4-fold higher than that of EG.5 and XBB.1.5,^1^ indicating that the low infectivity is not attribute the affinity to human ACE2. Yang et al. also analyzed the antigenic distance from B.1, BQ.1.1, BA.5, and XBB based on the neutralization titers of sera from the immunized mice with two doses of spike mRNA vaccines. They found that BA.2.86 was antigenically distinct from BA.5, BA.2, and XBB.1.5, suggesting that BA.2.86 significantly evades XBB-induced antibodies.^1^

Consequently, the researchers analyzed immune evasion of BA.2.86, one of the critical indicators for adopting health emergency measures. Uriu et al. and Sheward et al. demonstrated that therapeutic monoclonal antibodies (mAbs), such as bebtelovimab, sotrovimab, cilgavimab, and tixagevimab,^4,5^ showed weak neutralization activity against BA.2.86 pseudovirus infection. Yang et al. revealed similar results, but SA55 could still neutralize BA.2.86 efficiently.^1^ Compared with B.1.1 and BA.2, BA.2.86 is significantly resistant to the immunized sera of third-dose or fourth-dose monovalent, BA.5 bivalent, or BA.1 bivalent mRNA vaccine. However, even though the 50% neutralization tier (NT50) of sera from individuals with XBB breakthrough infection against BA.2.86 was significantly reduced, the titer was still 4- ~6-fold higher than that of sera from pre-XBB infected individuals.^5^ Sheward et al. demonstrated that the sera collected before the XBB wave exhibited about 4-fold lower NT50 against BA.2.86 than sera from XBB-infected persons.^4^ Yang et al. demonstrated that convalescent plasma from the XBB infection upon BF.7 or BA.5 breakthrough infection wave, showed higher NT50 against XBB.1.5, EG.5, and BA.2.86 than that from only XBB breakthrough infection, suggesting that multiple infection with Omicron variants may have an additive effect on the neutralizing activity of sera against BA.2.86.^1^ Wang et al. found that BA.2.86 exhibited higher sensitivity to neutralization by the sera from the “XBB breakthrough infection” cohort than those from the “BA.2 breakthrough infection” cohort. Most importantly, sera from the “XBB breakthrough infection” cohort could neutralize authentic BA.2.86 and EG.5.1 infection with the similar neutralization ID_50_ (50% inhibitory dilution).^2^ In sum, although BA.2.86 exhibited significantly more resistance to sera than other variants, XBB breakthrough infection or immunization with the latest COVID-19 booster vaccines could strengthen the preventive effect against BA.2.86 infection.

Nowadays, people in 154 countries and regions have been infected or reinfected with XBB variants, and some populations have already been vaccinated with the latest COVID-19 booster vaccines. As viral evolutionary biologist Jesse Bloom said, “I don’t think anybody needs to be alarmed by this, the most likely scenario is that this variant fizzles out”.^6^ Therefore, owing to widespread immunity, by vaccination and/or reinfection, against the Omicron variant and sublineages, BA.2.86 may not possess transmissibility on a global scale in the same proportion as previous Omicron variants.

Nonetheless, most studies on BA.2.86 have, thus far, were performed using pseudoviruses, not authentic viruses; therefore, the results may not be a true representation of NT50 values based on authentic virus infection. It is worth noting that BA.2.86 still keeps evolution, and it consists of eight different sublineages, namely BA.2.86, BA.2.86.1, BA.2.86.2, BA.2.86.3, JQ.1, JN.3, JN.2 and JN.1. As of October 31, 2023, North America and Europe have uploaded the majority of BA.2.86 sequences in GISAID (Fig. 1d). The predominant sublineage circulating in these regions is BA.2.86.1, although JN.1 is gradually increasing in prevalence (Fig. 1e, f). Compared to the reference sequence of BA.2.86 in Denmark and Israel, the S protein has undergone some new mutations, including L455S, P1263L, and V445Y. We cannot rule out the emergence of novel sublineages with higher infectivity and pathogenicity in the future. Therefore, we should continuously and comprehensively monitor the evolution of BA.2.86 and prepare accordingly against the next threats.
